# The Effects of Changing Attention and Context in an Awake Offline Processing Period on Visual Long-Term Memory

**DOI:** 10.3389/fpsyg.2015.01902

**Published:** 2016-01-07

**Authors:** Timothy M. Ellmore, Anna Feng, Kenneth Ng, Luthfunnahar Dewan, James C. Root

**Affiliations:** ^1^Department of Psychology, The City College of New York, City University of New YorkNew York, NY, USA; ^2^Program in Behavioral and Cognitive Neuroscience, The Graduate Center, City University of New YorkNew York, NY, USA; ^3^Department of Psychiatry and Behavioral Sciences, Memorial Sloan Kettering Cancer CenterNew York, NY, USA

**Keywords:** offline processing, attention, context, consolidation, intermediate memory, working memory, recognition memory

## Abstract

There is accumulating evidence that sleep as well as awake offline processing is important for the transformation of new experiences into long-term memory (LTM). Yet much remains to be understood about how various cognitive factors influence the efficiency of awake offline processing. In the present study we investigated how changes in attention and context in the immediate period after exposure to new visual information influences LTM consolidation. After presentation of multiple naturalistic scenes within a working memory paradigm, recognition was assessed 30 min and 24 h later in three groups of subjects. One group of subjects engaged in a focused attention task [the Revised Attentional Network Task (R-ANT)] in the 30 min after exposure to the scenes. Another group of subjects remained in the testing room during the 30 min after scene exposure and engaged in no goal- or task-directed activities. A third group of subjects left the testing room and returned 30 min later. A signal detection analysis revealed no significant differences among the three groups in hits, false alarms, or sensitivity on the 30-min recognition task. At the 24-h recognition test, the group that performed the R-ANT made significantly fewer hits compared to the group that left the testing room and did not perform the attention ask. The group that performed the R-ANT and the group that remained in the testing room during the 30-min post-exposure interval made significantly fewer false alarms on the 24-h recognition test compared to the group that left the testing room. The group that stayed in the testing room and engaged in no goal- or task-directed activities exhibited significantly higher sensitivity (*d*′) compared to the group that left the testing room and the group that performed the R-ANT task. Staying in the same context after exposure to new information and resting quietly with minimal engagement of attention results in the best ability to distinguish old from novel visual stimuli after 24 h. These findings suggest that changes in attentional demands and context during an immediate post-exposure offline processing interval modulate visual memory consolidation in a subtle but significant manner.

## Introduction

The human capacity to store complex visual information in long-term memory (LTM) is vast and well documented. In an early notable set of experiments (Standing et al., 1970), subjects recognized 90% of pictures from a set of over 2500 stimuli with only 1 s of display at initial encoding after 30 min and 24 h. While the capacity of visual information in LTM is high, much less is understood about the temporal dynamics and cognitive factors influencing the transformation of visual information from short-term memory (STM) to LTM. The type of processing after learning certainly is one important modulator; it is known that sleep and napping after learning improve the consolidation (i.e., permanent storage) of information in LTM (Stickgold, 2013), and that humans exhibit deficits in the ability to form new memories without regular periods of sleep (Yoo et al., 2007).

Aside from sleep or a nap, simple rest or quiet wakefulness without being engaged in any goal- or task-related processing facilitates memory strengthening (Ellenbogen et al., 2007) as it allows for “offline processing” including, most importantly for declarative memory, the replay of hippocampal-cortical traces and strengthening of horizontal cortico-cortical connections (O'Neill et al., 2010). Through this process, one idea is that memories stabilize or become resistant to interference during rest or quiet wakefulness and then consolidate during sleep (Walker, 2005). Another idea is that part of the process of consolidation takes place during wakefulness and periods of relative inactivity after the acquisition of new information (Buzsaki, 1989; Frankland and Bontempi, 2005).

In more typical learning situations outside a controlled testing environment, periods of wakefulness following the acquisition of new information are not accompanied by inactivity, but rather by a plethora of different cognitive processes and other experiences. How other active cognitive processes and behaviors influence the offline persistence of memory traces is a question beginning to be addressed by cognitive neuroscientists (Peigneux et al., 2006; Tambini et al., 2010; Staresina et al., 2013), but much still remains to be learned about how different types of active wakefulness following new learning affects the stabilization and consolidation of memories. This is an important area for future research as understanding the variables influencing how memories are made permanent in everyday scenarios has important implications for developing more effective education and training strategies, not to mention informing the experimental design of future basic memory investigations.

In the present study, we tested the hypothesis that changing attention and spatial context during an awake offline processing period immediately following exposure to new visual stimuli would affect the consolidation of stimuli into LTM. We predicted that engaging attention on an unrelated task and switching context would impair scene recognition by reducing the opportunity and efficiency of offline processing during wakeful activity.

## Materials and methods

### Subjects

Data were obtained from a total of 41 subjects (mean 19.75 years old, STD 2.39, 16 males, 4 left-handed). Each subject provided informed consent and completed the present study, which was approved by the Institutional Review Board of the City College of New York Human Research Protection Program. Subjects were recruited through the Sona Systems scheduling system of the Psychology Department. Each student received course extra credit for a total of 2 h of participation over the course of 2 days.

### Design and apparatus

A mixed within- and between-groups design was used. Subjects completed cognitive tasks inside a sound-attenuated booth (IAC Acoustics) to minimize auditory and visual distractions. Both the memory and attention tasks were programmed in Superlab 5 (Cedrus Corporation). Before performance of the actual tasks, subjects were given short (less than 5 min) demonstration tasks, with different stimuli for the scene tasks, to ensure that they understood the task instructions. The stimuli in the memory tasks were naturalistic scenes in 24-bit color sampled from the SUN database (Xiao et al., 2010). Each scene was displayed on a 27-inch LED monitor with a refresh rate of 60 hertz and a screen resolution of 1920-by-1080. Participants sat 83.5 cm from the monitor and maintained stable viewing using a combined forehead/chin rest. Each scene measured 800-by-600 pixels on the screen, and from the subject's point of view occupied a horizontal viewing angle of 17.2° and a vertical viewing angle of 12.7°.

**Figure 1:**
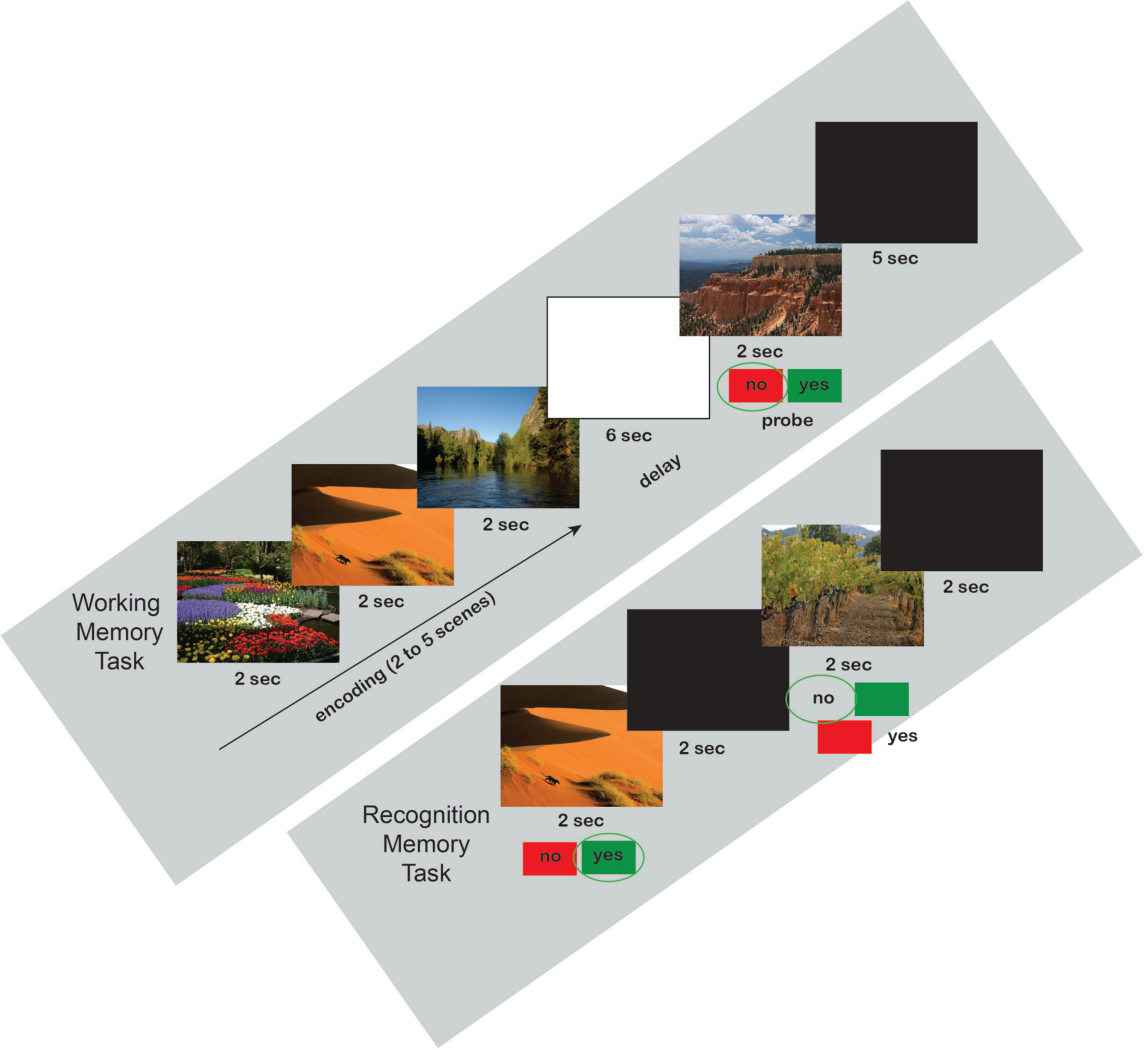
**Working Memory and Recognition Memory Scene Task Timelines**. All three groups performed the same working and recognition memory tasks. Examples are shown for a load = 3 working memory trial (above) with a negative probe, and the first two trials of a recognition memory test (below).

On the first day, each participant was exposed to a set of scenes by completing a 40-trial Sternberg working memory (WM) paradigm (see Figure 1 for example task timeline). Each trial consisted of a presentation of two to five scenes, each scene lasting 2 s (10 trials for each of the 4 loads). A 6 s blank screen delay period followed the presentation of the set of scenes. After the delay, a probe stimulus was presented for 2 s and, if the stimulus was considered to belong to the previously presented set (50% chance), the subject was required to press the right (green) button on a RB-530 response pad (Cedrus Inc.); if the probe did not belong to the previous set, the subject was required to press the left (red) button on the response pad. The scenes were presented within a modified Sternberg WM paradigm, rather than requiring subjects to passively view them. Following participation in the WM task, subjects were randomly assigned to one of the three groups. One group spent the following 30 min performing the revised version (Fan et al., 2009) of the Attentional Network Task (R-ANT) starting immediately after completion of the WM task.

In the R-ANT task, the subject is asked to determine the direction of the center arrow in a flanker array that may appear either to the left or right of fixation. The target is preceded by one of three cue conditions: *no cue*—no cue is presented; *double cue*—both cue boxes flash briefly to indicate when but not where the target will appear; *valid cue*—the left or right cue box flashes when and where the target will appear; *invalid cue*—the opposite cue box from where the target will appear flashes. Two conditions of flanker targets are included: *congruent*—all arrows point in the same direction; and *incongruent*—the center arrow points in the opposite direction from that of the flankers. These conditions allow an attention network to be identified through component decomposition. An alerting component is calculated by subtraction of the *no cue* condition from the *double cue* condition; an orienting component is calculated by subtraction of the *double cue* condition from the *valid cue* condition; and the executive component is calculated by subtraction of the *incongruent* target condition from the *congruent* target condition. The alerting component represents the earliest attention-related process and reflects arousal. The orienting component represents attentional shifting. The executive component represents the resolution of conflicting stimulus information. Subjects in the second group (No R-ANT, leave testing room) were dismissed from the testing room and told to return in 30 min; subjects in the third group (No R-ANT, stay in testing room) remained seated quietly in the testing room and did not engage in any goal-directed or task-related activity. None of the subjects in any of the three groups was told that there would be a recognition test after 30 min.

A total of 21 subjects who completed the R-ANT task after the scene WM task included 10 males, 2 left handed, with mean age 19.29 (STD 2.37). A total of 10 subjects who did not complete the R-ANT task and instead left the testing room for 30 min after completing the scene WM task included 3 males, 1 left handed, mean age 19.8 (STD 2.04). Another 10 subjects who did not complete the R-ANT task and instead remained in the testing room for 30 min after completing the scene WM task included 3 males, 1 left handed, mean age 20.6 (STD 2.72).

Following the 30-min interval, subjects in all three groups completed a 56 trial recognition memory test in which 30 randomly selected scenes from the WM task were randomly intermixed with 26 new scene images. Subjects were required to press the right (green) button if they thought they had already seen the scene (50% chance, with old scenes sampled from the WM task presentation), and the left (red) button if they decided that they had not seen the image (50% chance, new scenes sampled from SUN database). Scenes were presented one at a time for 2 sec. After the 30-min recognition test, each subject was told to return the following day at the same time. None of the subjects were informed on the nature of the test taking place the following day.

On the second testing day, all subjects completed a new 56-trial recognition memory test in which 26 of the stimuli had already been presented in the previous WM test. Importantly, none of the old stimuli presented during the 24-h recognition test was presented during the recognition test on the first day of testing; none of the novel stimuli presented during the 24-h recognition test was presented previously. Subjects were required to press the right (green) button if they thought they had already seen a stimulus (50% chance, with old stimuli sampled from the WM task presentation), and the left (red) button if they decided that they had not seen a stimulus previously (50% chance, new stimuli sampled from SUN database).

Also, on the second day of testing subjects who completed on the previous day the R-ANT task and subjects who remained in the testing room and engaged in no task completed a 14 item questionnaire of demonstrable reliability (Ellis et al., 1981) in which they rated the quality and duration of their previous night's sleep. The sleep ratings were only obtained for 10 of the subjects who during the previous day remained in the testing room and performed the R-ANT task and the 10 subjects who during the previous day remained in the testing room and did not complete the R-ANT task.

### Analysis

Performance across the forty WM trials was computed as percent correct to ensure that subjects were viewing the scenes and making decisions with a high level of accuracy. Percent correct accuracy was also computed for the R-ANT task for subjects who completed it between WM and recognition testing on day 1. A signal detection analysis (Stanislaw and Todorov, 1999) was performed on the 30 min and 24 h recognition task data. A hit was counted when a previously presented stimulus was signaled by the subject pressing a button indicating, correctly, that the stimulus had been previously seen (an old stimulus correctly classified as old). A false alarm was counted when a (new) stimulus not previously presented was indicated by the subject pressing a button indicating, incorrectly, that the image had been previously presented (a new stimulus incorrectly classified as an old stimulus). Total hits and false alarms were expressed as proportions in each subject and used to compute a measure of sensitivity as the difference in standardized normal deviates of hits minus false alarms: *d*′ = Z(hit rate) — Z(false alarm rate). The proportion of hits, false alarms, and sensitivity measures were analyzed using a mixed-design ANOVA in SPSS (v.21) with a within-subjects factor of time (time 0: working memory, time 1: recognition after 30 min, time 2: recognition after 24 h) and a between-subject factor of group (R-ANT task, leave testing room, stay in testing room). Paired comparisons of the proportion of hits, false alarms, and *d*′ for the 30 min and 24 h recognition tasks were compared using independent sample t-tests also in SPSS among subjects who performed the R-ANT task, the subjects who left the testing room for the 30 min between the WM and recognition test administered on the first day of testing, and the group that remained in the testing room for the 30 min between the WM and the recognition task administered on the first day of testing. *Post hoc* statistical power expressed, as 0 to 100%, for paired comparisons was computed according to (Rosner, 2011),

$$\begin{array}{l} Power=\Phi \left\{-Z_{1-\alpha ∕2}+\frac{\left|\mu_{2}-\mu_{1}\right|}{\sqrt{\left(\sigma_{1}^{2}∕n_{1}\right)}+\left(\sigma_{2}^{2}∕n_{2}\right)}\right\} \end{array}$$

where *n* is the sample size for a given group, μ is the group mean, σ is the variance of the mean, α is the probability of type I error (0.05), z is the critical *Z* value for α, and Φ is the function converting a critical *Z* value to power.

## Results

### Mixed-model ANOVA

For the analysis of hits, Mauchly's test indicated the assumption of sphericity had not been violated [X^2^_(2)_ = 1.24, *p* = 0.54], therefore the degrees of freedom were not corrected for the main effect of time [*F*_(2, 76)_ = 117.85, *p* < 0.0001, η${}_{\text{p}}^{2}$ = 0.76], the effect of group [*F*_(2, 38)_ = 0.37, *p* = 0.69, η${}_{\text{p}}^{2}$ = 0.02], or the interaction between time and group [*F*_(4, 76)_ = 1.78, *p* = 0.14, η${}_{\text{p}}^{2}$ = 0.09].

For the analysis of false alarms, Mauchly's test indicated no violation of the sphericity assumption [X^2^_(2)_ = 0.98, *p* = 0.71], so the degrees of freedom were not corrected for the main effect of time [*F*_(2, 76)_ = 20.04, *p* < 0.0001, η${}_{\text{p}}^{2}=0.34$], the effect of group [*F*_(2, 38)_ = 1.81, *p* = 0.18, η${}_{\text{p}}^{2}=0.09$], or the interaction between time and group [*F*_(4, 76)_ = 1.82, *p* = 0.13, η${}_{\text{p}}^{2}=0.09$].

For the analysis of *d*′ (sensitivity), Mauchly's test indicated no violation of sphericity [X^2^_(2)_ = 0.95, *p* = 0.42], so the degrees of freedom were not corrected for the main effect of time [*F*_(2, 76)_ = 179.80, *p* < 0.0001, η${}_{\text{p}}^{2}=0.83$], the effect of group [*F*_(2, 38)_ = 0.66, *p* = 0.52, η${}_{\text{p}}^{2}=0.03$], or the interaction between time and group [*F*_(4, 76)_ = 0.93, *p* = 0.45, η${}_{\text{p}}^{2}=0.05$].

### Exposure to scenes

Subjects in all three groups performed with similar accuracy on the WM task. Subjects assigned to leave the testing room (different context, no attention task) achieved 94.00% accuracy (STD 3.76) on the WM task. Subjects assigned to remain in the testing room (same context, no attention task) performed at 93.75% (STD 4.89) on the WM task. Subjects assigned to remain in the testing room and perform the R-ANT task during the retention interval (same context, attention task) performed at 93.93% (STD 4.23) on the WM task. The subjects who stayed in the testing room and performed the R-ANT task completed the R-ANT with an accuracy of 96.92% (STD 2.74).

### Recognition after 30 min

**Figure 2:**
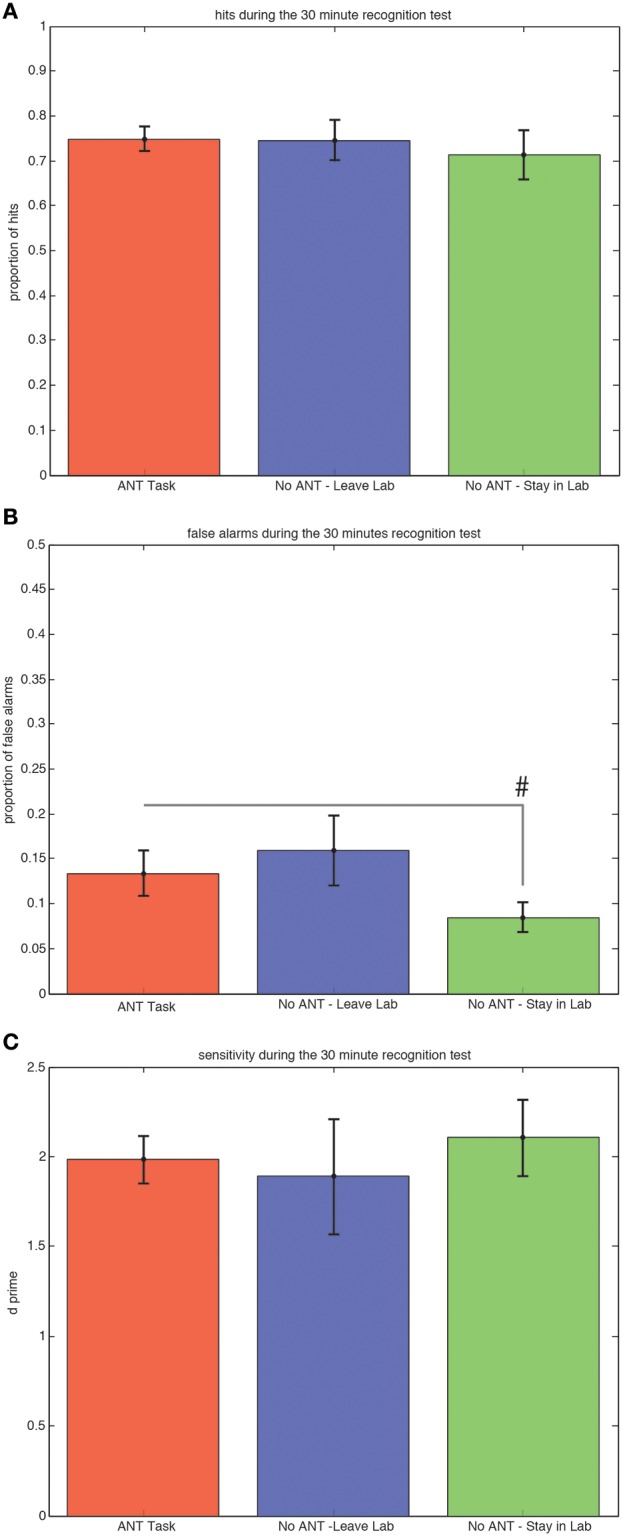
**Ability to Discriminate Old from New Scenes at the 30 min Recognition Test is Not Affected by Changing Attention or Context during Awake Offline Processing**. Average hits (± SEM) do not differ among groups **(A)**. There is a trend (#*p* < 0.1) for subjects who remained in the testing room to make fewer false alarms compared to subjects who performed the R-ANT **(B)**. There is no difference in sensitivity **(C)** among the three groups.

There were no significant differences among the three groups of subjects in either the proportion of hits (Figure 2A) or sensitivity (*d*-prime, Figure 2C) during the 30-min recognition test. Subjects who stayed in the testing room and did not engage in any task during the 30 min interval made fewer false alarm responses compared to subjects who stayed in the testing room and completed the R-ANT task (Figure 2B) but this tendency did not reach the *a priori* threshold for statistical significance [0.08 versus 0.13, *t*_(29)_ = 1.32, *p* = 0.10, STD error of difference = 0.04, power = 39.5%].

### Recognition after 24 h

**Figure 3:**
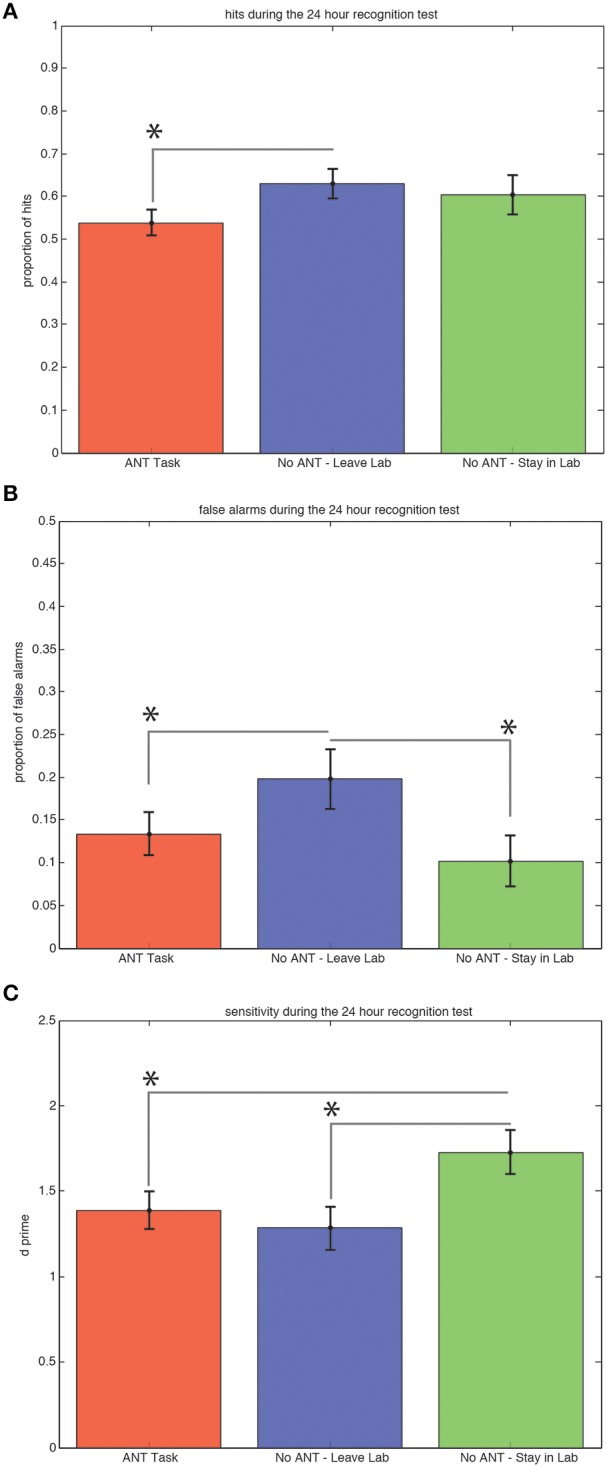
**Ability to Discriminate Old from New Scenes at the 24 h Recognition Test is Better for Subjects Who Remained in the Same Context and Rested Quietly**. Average hits (± SEM) are significantly lower for the group that performed the R-ANT task **(A)**. Subjects who left the testing room made significantly (^*^p < 0.05) more false alarms **(B)** compared to subjects who performed the R-ANT task and subjects who remained in the testing room and rested quietly. Subjects who stayed in the testing room and rested quietly showed significantly higher sensitivity compared to the subjects who performed the attention task and those who changed spatial context by leaving the testing room during the 30-min post-exposure period **(C)**.

Subjects who left the testing room during the 30-min interval after exposure to the scenes on the first day obtained significantly more hits (Figure 3A) during the recognition test 24 h later compared to subjects who stayed in the testing room and performed the task [0.63 versus 0.54, *t*_(29)_ = 1.91, *p* = 0.03, STD error of difference = 0.05, power = 54.5%].

Subjects who left the testing room during the 30-min interval after exposure to the scenes on the first day also made significantly more false alarms (Figure 3B) during the recognition test 24 h later compared to both the subjects who stayed in the testing room and performed the R-ANT task [0.20 versus 0.13, *t*_(29)_ = 1.67, *p* = 0.05, STD error of difference = 0.04, power = 40.3%] and compared to the subjects who remained in the testing room and did not perform the task [0.20 versus 0.10, *t*_(18)_ = 2.24, *p* = 0.02, STD error of difference = 0.04, power = 61.3%].

Subjects who remained in the testing room and did not perform the R-ANT task during the 30-min interval after exposure to the scenes on the first day exhibited the highest sensitivity *(d*′*)* during the recognition test 24 h later (Figure 3C) compared to subjects who left the testing room [1.73 versus 1.28, *t*_(18)_ = 2.52, *p* = 0.03, STD error of difference = 0.18, power = 71.1%] and also compared to subjects who remained in the testing room and performed the R-ANT task [1.73 versus 1.39, *t*_(29)_ = 1.91, *p* = 0.03, STD error of difference = 0.18, power = 53.3%].

### Sleep duration and quality

Sleep duration and quality for the night between day 1 scene exposure and day 2 recognition testing indicated no differences between subjects who remained in the testing room and performed the R-ANT testing and subjects who remained in the testing room and did not complete the R-ANT task [self-reported sleep quality from 1 “very light” to 8 “very deep”: mean 5.30 ± 1.49 (STD) versus 5.62 ± 1.77, independent sample t-test *p* = 0.68; self-reported number of times awoke during the night 0 “not at all” to 7 “more than 6 times”: 0.60 ± 0.84 versus 0.50 ± 0.76, *p* = 0.80; number self-reported hours of sleep: 6.77 ± 1.60 versus 6.16 ± 1.75, *p* = 0.44].

## Discussion

Entering a sleep state makes it more likely that information encountered during the immediately preceding awake period will be remembered (Walker, 2005; Stickgold, 2013). The beneficial effect of sleep for memory consolidation is hypothesized to depend in part on offline reprocessing of the information to create a stronger memory trace. Offline processing does not just occur during sleep; it is also thought to occur during awake periods, like quiet rest (Tambini et al., 2010; Staresina et al., 2013; Vilberg and Davachi, 2013; Schlichting and Preston, 2014). In the present study we tested the hypothesis that changing cognitive demands in the awake period immediately after the exposure to information would affect LTM for that information. We studied three groups of subjects all of whom were exposed to the same set of naturalistic color scenes. In the 30 min immediately following the exposure, one group remained in the testing room and engaged in an attention demanding task; another group was dismissed from the testing room thereby experiencing a switch in the context for offline processing and told to return 30 min later with no constraints on the activities in which they spent the 30 min, as long as it was outside the testing room. A third group remained in the testing room and waited quietly, engaging in no particular task- or goal-directed activity.

All subjects in each group completed a recognition task after the post-exposure interval but no group showed any significant difference in hits, false alarms, or sensitivity to distinguish old from novel scenes during the 30-min recognition test. All participants came back 24 h later for another recognition test involving unique subsets of new scenes and old scenes encountered during the previous exposure on the first day. There were significant differences among the groups in the 24-h recognition performance. The group of participants who performed the attention task during the 30-min interval on the first day showed the poorest hit rate at the 24-h recognition test. The group that left the testing room during the 30-min interval on the previous day showed the highest false alarm rate, significantly higher than the group that performed the attention task and the group that remained in the testing room. The group that remained in the testing room and waited quietly and engaging in no particular task- or goal-directed activity demonstrated the highest sensitivity for distinguishing old from new scenes after 24 h. These results indicate that varying attentional engagement and the context in which awake offline processing occurs in the immediate period following exposure to novel visual stimuli affects LTM for those stimuli.

Engaging attention in an unrelated task immediately after exposure to new stimuli could reduce the efficiency of offline processing in a period of time that is critical to start the neurobiological process of memory trace replay thought necessary to consolidate information (Rosenzweig et al., 1993; McGaugh, 2000; Frankland and Bontempi, 2005; O'Neill et al., 2010). Switching the context from where the initial exposure to new information occurred could increase the likelihood of encountering similar visual stimuli, which could increase interference (Keppel and Underwood, 1962) thereby also decreasing the efficiency of offline processing. While it is plausible that the groups of subjects who underwent the attention focus and context switches experienced reduced efficiency of offline processing during the immediate post-exposure period, there are several caveats and limitations to this study that must be highlighted.

First, we exposed our subjects to the set of scenes by presenting them within a modified Sternberg WM paradigm (Sternberg, 1969). This task required subjects to keep these scenes in STM for a brief period and then make a decision about whether a probe matched one of the previously presented scenes. We hypothesized that this task would engage subjects more so than merely requiring them to passively view the scenes, but we did not have a passive viewing group in the present study test this assumption. While subjects in all of the groups tested performed the WM task above 90% accuracy, it is not possible to say definitively that subjects actually learned or encoded all of these scenes equally well. However, we obtained some evidence for learning because subjects identified previously presented scenes from the set well above chance on average (hit rate for all groups > 70%), and there were no differences across groups for the hit rates in the first recognition test administered after the 30 min interval.

Does offline processing in the awake state immediately after exposure to visual stimuli result in equally good LTM if a subject's attention is engaged in an unrelated task? We addressed this question by having one group of subjects perform the R-ANT task and compared their performance to a group of subjects who remained in the testing room and engaged in no task- or goal-oriented activity that demanded attention. In the R-ANT task, the subject is asked to determine the direction of the center arrow in a flanker array that may appear either to the left or right of fixation. On some trials the subject is given valid or invalid cues but these cues only last 100 ms before a 400 ms gap separating the presentation of the set of arrows. This rapid presentation requires subjects to pay attention, and completion of the dozens of trials over a half hour can be quite taxing especially if performance on the task is high, which it was on average (>90%) in the subjects we tested. The task consists of boxes, a fixation cross and sets of arrows, which are stimuli that are not complex and in no way resemble the color scenes which we used in the WM and recognition memory tasks. Therefore interference from the R-ANT stimuli should be low. Subjects who completed the R-ANT task and took the recognition memory task at 30 min performed equally as well as subjects who remained in the testing room and engaged in no attention tasks or goal-directed activity. When subjects who performed the R-ANT completed the 24-h recognition test, they exhibited reduced sensitivity *(d*′*)* for discriminating old from new scenes. This finding suggests that engaging attention in the intermediate period after exposure to new stimuli subtly but significantly impacts LTM at 24 h but not 30 min. To rule out differences in sleep between the two groups as a potential confound which could explain a difference in performance at 24 h but not 30 min, we administered a sleep questionnaire (Ellis et al., 1981) to subjects when they returned for the 24 h recognition test. We found no significant differences between those who performed the R-ANT task and those who remained in the testing room and waited quietly. We interpret these results as indicating that there is a slight advantage for visual LTM in allowing subjects to rest quietly in the awake state rather than occupy their time with a demanding albeit unrelated cognitive task.

We also conducted a comparison in a group of subjects who were allowed the opportunity to experience offline processing in an uncontrolled condition in which they were not allowed to remain in the testing room after exposure to the scenes. This group of participants experienced the 30 min between exposure to the scenes and the first recognition task *outside* the testing room in a different context, engaging in whatever activity they so desired. This condition is more ecologically valid, as offline processing after exposure to new stimuli often takes place in the real world in new contexts during highly variable cognitive conditions. Surprisingly even after experiencing such an uncontrolled condition, the different context group achieved similar hit rates, false alarm rates, and sensitivity at the 30 min recognition test. They did, however, show some significant differences at the 24 h recognition test, including higher false alarms compared to the group that performed the RANT task and compared to the group that remained in the testing room and did no task. They also showed reduced sensitivity *(d*′*)* compared to the group that remained in the testing room and did no task. The higher false alarm rate could be attributed to the opportunity for these subjects to encounter a highly variable set of other visual stimuli during the intervening 30 min, which could have interfered with the scene stimuli presented during the task. A major limitation of this study is that for this group we did not control precisely what these subjects saw or which other contexts they experienced outside the testing room. There is an opportunity for future studies to systematically vary the both exposure to similar stimuli (thereby increasing interference) and spatial context in a more controlled fashion by allowing subjects to experience offline processing in virtual worlds through the use of immersive virtual reality technology. Similar manipulations have proved extremely successfully in extending a basic understanding of how place, context, and temporal order are represented behaviorally and at the neural level (Burgess et al., 2001, 2002).

Finally, these are data from small samples of unequal groups of young adults. Our paired comparisons do not survive strict Bonferroni multiple comparisons corrections and *post hoc* power computations indicate variable power ranging from 40 to 70%. Therefore further study in a larger sample with additional controls will need to be conducted to understand why exactly focusing attention and changing context in the immediate post-learning period impacts LTM consolidation for visual information in a subtle but statistically significant manner.

Some neurobiological evidence implicates the hippocampus as orchestrating a process of memory trace replay that may, for some memories, strengthen horizontal connections among cortical areas (Buzsaki, 1989; Eichenbaum et al., 1994; Dudai et al., 2015). As a result of this process, the trace may be stabilized and consolidated into a more durable state (Frankland and Bontempi, 2005). Opportunities for reconsolidation or forgetting (Hardt et al., 2013) exist if the stimuli are re-experienced in the same or different context. The optimal time duration for offline processing is not known but may last from minutes to a day or more, with one or more periods of sleep thought to facilitate consolidation (Drosopoulos et al., 2005; Yoo et al., 2007). Our experimental manipulation of offline processing involved a relatively brief 30-min period after exposure to the novel visual information. Although we saw some significant effects among our groups at the 24-h recognition test, these effects were small and likely due to a combination of the small period of time in which we manipulated cognitive demands during the offline processing. Increasing the sample size, adding more tightly controlled cognitive constraints including a sleep or nap group, understanding more thoroughly the thought processes accompanying quiet wakefulness (Hurlburt et al., 2015) and combining the behavioral manipulations with electrophysiological monitoring like EEG (Huber et al., 2004) or neuroimaging (Tambini et al., 2010; Spadone et al., 2015) will help better understand the contribution of awake offline processing to memory consolidation.

## Conclusion

In conclusion, the present findings suggest that changes in the focusing of attention and context during offline processing in the minutes after exposure to novel visual stimuli modulate LTM consolidation in a subtle but significant way. During the offline processing period, remaining in the same context and resting quietly with minimal attention demands results in the best sensitivity for distinguishing old from novel visual stimuli after 24 h.

### Conflict of interest statement

The authors declare that the research was conducted in the absence of any commercial or financial relationships that could be construed as a potential conflict of interest. The reviewer Elisa Di Rosa and handling Editor Antonino Vallesi declared their shared affiliation, and the handling Editor states that the process nevertheless met the standards of a fair and objective review.
